# Ketogenic Diet Modulates NAD^+^-Dependent Enzymes and Reduces DNA Damage in Hippocampus

**DOI:** 10.3389/fncel.2018.00263

**Published:** 2018-08-30

**Authors:** Marwa Elamin, David N. Ruskin, Susan A. Masino, Paola Sacchetti

**Affiliations:** ^1^Graduate Program in Neuroscience, Department of Biology, University of Hartford, West Hartford, CT, United States; ^2^Neuroscience Program and Psychology Department, Trinity College, Hartford, CT, United States

**Keywords:** ketone bodies, metabolism, hippocampus, longevity, oxidative stress, nicotinamide adenine dinucleotide, sirtuin, PARP-1

## Abstract

The ketogenic diet’s (KD) anti-seizure effects have long been documented. Recently, its therapeutic potential in multiple neurodegenerative and neurodevelopmental disorders has emerged. Yet experimental evidence for a fundamental mechanism underlying beneficial effects across numerous diseases remains lacking. We previously showed that feeding rats a KD produced an early (within 2 days) and persistent elevation of hippocampal nicotinamide adenine dinucleotide^+^ (NAD^+^), an essential metabolic coenzyme and signaling molecule. NAD^+^ is a marker of cellular health and a substrate for enzymes implicated in longevity and DNA damage repair such as sirtuins and poly-ADP ribose polymerase-1 (PARP-1). As a result, activation of NAD^+^-dependent enzymes’ downstream pathways could be the origin of KD’s broad beneficial effects. Here rats were fed *ad libitum* regular chow or KD for 2 days or 3 weeks and the levels of hippocampal sirtuins, PARP-1, and the oxidative DNA damage marker 8-hydroxy-2’-deoxyguanosine were quantified. We found a significant immediate and persistent increase in the collective activity of nuclear sirtuin enzymes, and a significant augmentation of Sirt1 mRNA at 2 days. Levels of PARP-1 and 8-hydroxy-2’-deoxyguanosine decreased after 2 days of treatment and further declined at 3 weeks. Our data show that a KD can rapidly modulate energy metabolism by acting on NAD^+^-dependent enzymes and their downstream pathways. Thus, therapy with a KD can potentially enhance brain health and increase overall healthspan via NAD^+^-related mechanisms that render cells more resilient against DNA damage and a host of metabolic, epileptic, neurodegenerative, or neurodevelopmental insults.

## Introduction

The ketogenic diet (KD) is a high-fat, low-carbohydrate, moderate protein therapy that shifts energy production away from glucose-based and toward ketone-based ATP production. It is effective in treating pharmacoresistant epilepsy (Neal et al., 2009; Sharma et al., 2013; Cervenka et al., 2017) and growing evidence supports its beneficial effects in diverse disorders (Yang and Cheng, 2010; Winter et al., 2017; Augustin et al., 2018) and in healthspan and lifespan (Newman et al., 2017; Roberts et al., 2017). As the fundamental mechanism(s) underlying its success remain unclear, molecular changes induced by the diet must be characterized in healthy cells.

Recently we proposed that the broad effectiveness of the KD is related to increased levels of the coenzyme nicotinamide adenine dinucleotide (NAD) (Elamin et al., 2017), a pivotal molecule for redox reactions and the backbone of ATP generation. Because fewer NAD molecules are reduced during ketone-based vs. glucose-based metabolism in brain, elevated levels of the oxidized form (NAD^+^) can be expected. Consistent with this prediction, we demonstrated that KD can induce rapid and sustained changes in the NAD^+^/NADH ratio in rat hippocampus - an energetically demanding brain region considered a seizure gate (Heinemann et al., 1992) - but not in the cerebral cortex (Elamin et al., 2017). Similar results have been found in aged mice with a ketone ester supplemented diet (Pawlosky et al., 2017).

Elevated NAD^+^ limits seizures and mediates lifespan extension (Lin and Guarente, 2003; Mills et al., 2016; Liu et al., 2017). Experimentally increased NAD^+^ levels enhance mitochondrial function, protect against oxidative stress damage and decrease cell death (Kussmaul and Hirst, 2006), diverse effects that could be linked to downstream pathways. NAD^+^ serves as a substrate for two enzyme groups, sirtuins and poly(ADP-ribose) polymerases (PARPs), that affect diverse cellular functions ranging from gene expression to DNA repair (Belenky et al., 2007).

The NAD^+^-dependent sirtuin enzymes are important regulators of metabolism, inflammation, and DNA repair (Michishita, 2005). Sirt1 is involved in the deacetylation of transcription factors, growth factors, anti-apoptotic, and anti-inflammatory proteins (Yang et al., 2006). Sirt1 is essential for normal cognition, preservation of memory and neuronal plasticity (Michan et al., 2010). Interestingly, Sirt1 has seizure-suppressing effects in animal models of epilepsy (Wang et al., 2016) and mediates benefits of caloric restriction, such as lifespan extension and promotion of cell survival (Lin et al., 2000; Cohen et al., 2004; Satoh et al., 2013). Sirt6 and Sirt7 participate directly in base-excision repair of DNA, thus decreasing age-associated DNA damage (Mostoslavsky et al., 2006; Vazquez et al., 2016).

PARP enzymes add polymers of ADP-ribose to nuclear proteins in an NAD^+^-dependent matter and are major consumers of NAD^+^. PARP-1 plays an important role in cell survival and DNA damage repair (Grube and Burkle, 1992; de Murcia and de Murcia, 1994). DNA oxidation by reactive oxygen species induces a steady-state level of DNA damage and is a by-product of normal cellular metabolism. PARP-1 is a molecular sensor for this type of DNA damage and both its activity and protein levels are affected directly by cellular levels of oxidative DNA damage (Dantzer et al., 2006; Bürkle and Virág, 2013; Shen et al., 2016). Although PARP enzymes play a vital role in DNA repair, their over-activation (and subsequent depletion of NAD^+^) has been linked to diverse neuropathological conditions (Morales et al., 2014; Martire et al., 2015).

Since the KD rapidly increases NAD^+^ levels (Elamin et al., 2017) and changes in NAD^+^ levels modulate the activity of the abovementioned enzymatic pathways, we hypothesized that consumption of a KD would lead to downstream beneficial changes in NAD^+^-dependent enzymes’ activity in rat hippocampus.

## Materials and Methods

### Animals and Dietary Treatment

Sprague-Dawley male rats (age 11–14 week; 350–550 g; *n* = 22) were pair-housed at Trinity College, with water and food *ad libitum*. Animals received either a high-carbohydrate chow diet (Purina 5001, PharmaServ, Framingham, MA, United States) (CD), or a 6:1 (fat:protein+carbohydrates) KD (F3666, Bio-Serv, Frenchtown, NJ, United States) for 2 days (2d) or 3 weeks (3w); (*n* = 8 each). At the end of dietary treatment, animals were sacrificed, trunk blood was collected and the hippocampi were dissected. Plasma β-hydroxybutyrate was measured using Precision Xtra monitors (Abbott Laboratories; Abbott Park, Chicago, IL, United States). All experiments were in compliance with National Institutes of Health Guides and Trinity College Animal Care and Use Committee.

### NAD^+^/NADH Analysis

Analysis was done according to manufacturer’s instructions (Sigma-Aldrich, United States) as previously described (Elamin et al., 2017). Optical density of NAD^+^/NADH and NADH were obtained at 450 nm. NAD^+^ values were calculated by subtracting NADH values from total NAD values and normalized to protein concentrations.

### Sirtuin Activity

Hippocampal tissues were homogenized in PBS lysis buffer [137 mM NaCl, 2.7 mM KCl, 10 mM Tris pH 7.4, 1 mM PMSF, 1:1000 Protease inhibitor cocktail (PIC)]. Pellets were suspended in Extraction lysis buffer (20 mM Tris pH 7.8, 125 mM NaCl, 5 mM MgCl_2,_ 0.2 mM EDTA, 0.1% NP40, 12% glycerol, 200 mM PMSF, 200 mM DDT, 1:1000 PIC) and sonicated. After centrifugation, combined activity of nuclear sirtuin enzymes (Sirt1,6,7) was measured in hippocampal nuclear extracts following manufacturer’s instruction (Epigentek, United States).

### Real-Time PCR

Total hippocampal RNA (1 μg) was extracted (Qiagen, United States), reverse transcribed (Applied Biosystems, United States), and relative mRNA levels were detected by quantitative PCR (StepOnePlus, Thermo Fisher, United States). Predesigned rat Sirt1, Sirt6, Sirt7, and β-actin TaqMan Gene Expression probes (Assay IDs: Rn01428096_m1, Rn01408249_m1, Rn01471420_m1, and Rn00667869_m1, respectively) were used with TaqMan Gene Expression Master Mix (Thermo Fisher, United States). Analyses were performed using the standard curve method with Sirt transcripts normalized to β-actin as the endogenous control.

### Western Blot

Hippocampal homogenates made in RIPA buffer were analyzed by western blot for PARP-1 (Cell Signaling) and β-actin (Neomarkers) proteins (1:1000 primary antibodies). Blots were revealed by Chemiluminescence (Thermo Fisher, United States) and quantified using ImageJ software (National Institutes of Health, Bethesda, MD, United States).

### DNA Damage Analysis

Purified DNA was obtained from hippocampal cells (DNeasy Blood and Tissue kit, Qiagen, United States) and levels of 8-OHdG (8-hydroxy-2′-deoxyguanosine) were quantified by competitive ELISA assay (StressMarq, United States) following manufacturer’s protocol.

### Statistical Analysis

One-way ANOVA and *post hoc* Tukey’s multiple comparisons test were performed for all experiments using GraphPad Prism software (GraphPad, United States). Data are expressed as mean ± SEM representing the average of two measurements per sample using the animal number indicated as subject number. ^∗∗^*P* < 0.01, ^∗∗∗^*P* < 0.001, ^∗∗∗∗^*P* < 0.0001, non-significant (ns).

## Results

**FIGURE 1:**
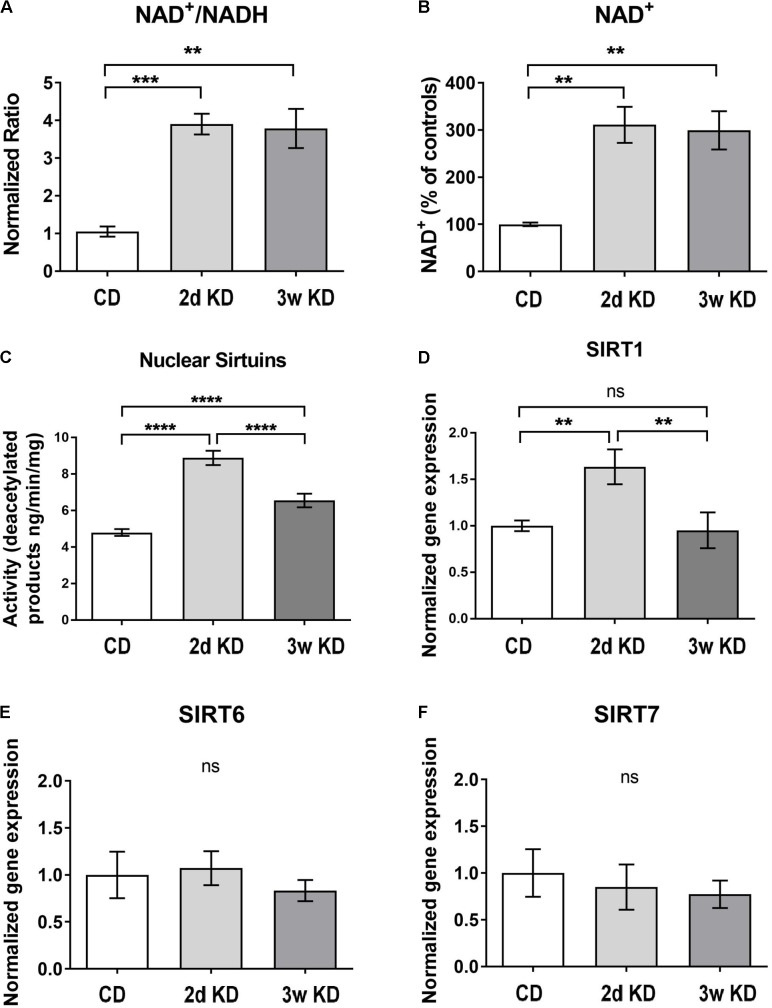
Hippocampal changes in NAD and sirtuins after ketogenic diet (KD) treatment. **(A,B)** NAD^+^/NADH ratios and NAD^+^ levels in the hippocampus after standard chow diet (CD; *n* = 5), 2 day (2d; *n* = 7), or 3 weeks KD treatment (3w; *n* = 7). **(C)** Collective deacetylation activity of Sirt1, Sirt6, and Sirt7 enzymes. A significant increase was observed at 2d and remained elevated at 3w. *n* = 6–8 animals (*n* = 2 per animal). **(D–F)** Real-time PCR analysis of Sirt1,–6,–7 gene expression in hippocampus. Sirt1 expression was increased only at 2d of treatment. CD, *n* = 6; 2d KD, *n* = 8; 3w KD, *n* = 8 (*n* = 2 per animal).

To re-establish previous observations on rapid and sustained KD-induced increases in NAD^+^ availability (Elamin et al., 2017), we treated a new cohort of rats with control diet or KD. In addition to replicating increased circulating ketones (mM: 0.76 ± 0.15 2d KD, 0.67 ± 0.23 3w KD, 0.05 ± 0.02 CD; *p* = 0.0189) and NAD^+^/NADH ratios in response to KD (**Figure 1A**), we established that the increase in ratio was solely due to an increase in the oxidized form of NAD (**Figure 1B**).

To address downstream effects sirtuin enzymes’ activity, and expression, which depend directly on the levels of the main substrate NAD^+^ (Landry et al., 2000; Chen et al., 2008), were examined and the collective deacetylation activity of nuclear sirtuin enzymes (Sirt1, Sirt6, Sirt7) was measured in hippocampal nuclear extracts. As shown in **Figure 1C**, a rapid, robust increase in sirtuins’ activity was detected at 2d of KD treatment compared to control, and remained elevated at 3w, albeit reduced. Changes in enzymatic activity could be reflective of alterations in gene expression of one or more of these enzymes. Analysis of nuclear sirtuins expression levels showed that Sirt1 mRNA was increased in animals fed a KD for 2d compared to control diet but normalized after 3w of treatment (**Figure 1D**). No significant mRNA changes were observed for Sirt6 and Sirt7 (**Figures 1E,F**).

**FIGURE 2:**
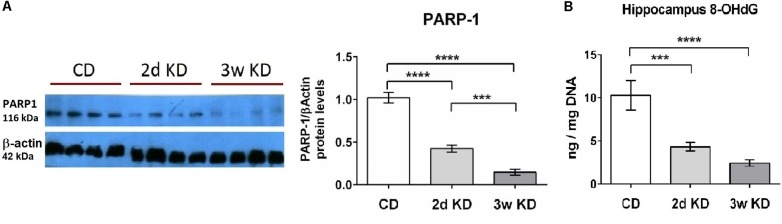
Effect of KD treatment on hippocampal PARP-1 levels and 8-OHdG levels. **(A)** Quantification of normalized PARP-1 protein levels in hippocampi obtained from animals fed standard chow (CD), or KD for 2d or 3w. Representative image of PARP-1 Western blot included. Each lane represents an individual animal. Blots were repeated 2–3 times. **(B)** Significant and progressive decrease in 8-OHdG levels was observed in hippocampi obtained from KD treated animals. CD, *n* = 6; 2d KD, *n* = 8; 3w KD, *n* = 8 (*n* = 2 for each animal).

NAD^+^ also serves as main substrate for PARP-1, an enzyme that functions as a DNA damage sensor and participates in DNA repair. We, therefore, examined potential changes in the hippocampal DNA damage response after KD treatment. A rapid and dramatic decline in PARP-1 protein level was detected after 2d, with further reduction upon longer KD exposure (**Figure 2A**). Although PARP-1 is a major consumer of NAD^+^, the activity and protein levels of this enzyme are modulated primarily by levels of oxidative DNA damage (Dantzer et al., 2006; Shen et al., 2016). Therefore DNA damage in hippocampal tissue was assessed further by quantification of 8-OHdG, a critical biomarker of oxidative stress. Dietary treatment with 2d KD decreased the levels of 8-OHdG, and a 3w treatment led to a further decrease in this biomarker (**Figure 2B**). PARP-1 protein levels strongly correlated with the changes in oxidative DNA damage here detected (Martinet et al., 2002; Huber et al., 2004). Together, observed decreases in PARP-1 and 8-OHdG levels suggest that consuming a KD decreases oxidative DNA damage.

## Discussion

Here we determined that KD increased NAD^+^, decreased levels of DNA damage and induced rapid changes in PARP-1 and sirtuin enzymes. This cohort of changes induced within 2 days of KD exposure could thus be protective for healthy cells against oxidative and metabolic damage and provide key mechanisms rendering KD beneficial across a range of neurological conditions.

The time-course of changes in Sirt1 activity and expression parallel the persistent increase in NAD^+^ levels (Elamin et al., 2017) and support the idea that elevated NAD^+^ increases activation of sirtuin enzymes. The ability of Sirt1 to affect the expression of genes implicated in a variety of functions ranging from neuroinflammation to proliferation and apoptosis (Cheng et al., 2003; Jeong et al., 2011) could explain the KD’s ability to impact diverse cellular pathways. Sirt1 activity reduces cell death and inflammation (Yeung et al., 2004; Kauppinen et al., 2013) and increases neuronal survival and life-span (Cohen et al., 2004; Kim et al., 2007; Khan et al., 2012). Therefore beneficial effects of ketogenic treatment in decreasing inflammation and combating neurodegenerative diseases could be attributed to these molecular mechanisms (Gasior et al., 2006; Ruskin et al., 2009; Kashiwaya et al., 2013).

Several genes controlling ketosis, fatty acid oxidation, and mitochondrial biogenesis are upregulated after long periods of KD treatment (Cullingford et al., 2002; Bough et al., 2006; Kennedy et al., 2007). Augmented sirtuin enzymatic activity without enhanced gene expression observed at 3 weeks may be due to elevated levels of the substrate NAD^+^. As Sirt1 was found to play a role in hepatocellular lipid metabolism (Hou et al., 2008), the transient upregulation of Sirt1 could be a cellular response to the shift in energy metabolism from glucose to fatty acids and disappear after adaptation occurs.

Previous work illustrated that ketogenic treatment reduced reactive oxygen species (Maalouf et al., 2007; Greco et al., 2016). Notably, Sirt1 can prevent neuronal cell death by reducing oxidative stress (Khan et al., 2012; Singh et al., 2017) which has been implicated in several neurodegenerative disorders (Andersen, 2004; Guo et al., 2013; Jiang et al., 2016; Islam, 2017). A consequence of oxidative stress and impaired mitochondrial function is DNA damage (Beal, 1995). This type of oxidative damage occurs as a by-product of normal cellular respiration, and can significantly contribute to the process of aging and neurodegeneration (Yakes and Van Houten, 1997). Moderate DNA damage triggers PARP-1 activation and DNA repair, resulting in reduced 8-OHdG levels (Hegedűs and Virág, 2014). Our quantified decrease in 8-OHdG and correlated PARP-1 changes suggest that ketogenic therapy can also rapidly and directly modulate DNA repair and oxidative stress. Therefore, potential direct effects, as well as elevation of Sirt activity and/or NAD^+^ inhibiting DNA damage (Hou et al., 2018) and decreasing reactive oxygen species (Lin and Guarente, 2003; Barzilai and Yamamoto, 2004; Kussmaul and Hirst, 2006) could contribute to decreased DNA damage. Interestingly, inhibition of PARP-1 can enhance mitochondrial metabolism and activate Sirt1 enzyme (Bai et al., 2011). Thus our data reinforce the existence of cross-talk between Sirt1 and PARP-1 (Cantó et al., 2013), possibly triggered by KD treatment, and modulated by NAD^+^ levels (Fouquerel and Sobol, 2014; Hegedűs and Virág, 2014; Mendelsohn and Larrick, 2017).

The rapid response of NAD^+^ and downstream effectors may relate to the anti-seizure response found in some patients after just a few days of KD treatment (Freeman and Vining, 1999; Cervenka et al., 2017). Moreover, elevation in PARP-1 activity, accompanied by NAD^+^ depletion and reduced Sirt1 activity, was shown to mediate neuronal death after seizure induction in animal models (Wang et al., 2013). Our data point to an opposite effect of KD on these three cellular measures, thus hinting at these effects as potential anti-seizure mechanisms. In addition, neuronal cell death and hippocampal 8-OHdG levels were increased following kainate-induced seizures in rats and prevented by administration of antioxidants (Liang et al., 2000). The KD’s ability to modulate oxidative DNA damage and reduce 8-OHdG levels in the hippocampus suggests that KD treatment may also be protective against seizure-induced neuronal death and DNA damage.

Here we quantified altered endogenous NAD^+^ levels and key downstream effectors as a direct result of consuming a KD and offered key potential mechanisms underlying anti-seizure and neuroprotective effects of ketogenic therapy. The changes found in disease-free animals in our current and previous studies (Elamin et al., 2017) indicate that the use of ketone bodies as an energy source can be associated with a healthier metabolic phenotype and an enhanced redox state even in the absence of disease or senescence. Furthermore, the NAD^+^ precursor nicotinamide ribose and PARP inhibitors are proposed to combat cancer and a host of inflammatory and neurodegenerative diseases (Basello and Scovassi, 2015; Lee et al., 2015; Ohmoto and Yachida, 2017; Vaur et al., 2017). We provide evidence that consuming a KD can mobilize similar mechanisms, and promote the metabolic resilience necessary to combat neurodegenerative and age-associated diseases.

## Author Contributions

ME, DR, SM, and PS conceived and designed the experiments, analyzed and interpreted the data, drafted the manuscript, and approved the final manuscript to be published. ME and DR acquired the data.

## Conflict of Interest Statement

The authors declare that the research was conducted in the absence of any commercial or financial relationships that could be construed as a potential conflict of interest.
